# Genomic and Transcriptomic Analyses Identify Two Key Glycosyltransferase Genes *alhH* and *alhK* of Exopolysaccharide Biosynthesis in *Pantoea alhagi* NX-11

**DOI:** 10.3390/microorganisms12102016

**Published:** 2024-10-05

**Authors:** Kun He, Xiaolong Shi, Zhongming Tao, Xing Hu, Liang Sun, Rui Wang, Yian Gu, Hong Xu, Yibin Qiu, Peng Lei

**Affiliations:** State Key Laboratory of Materials-Oriented Chemical Engineering, College of Food Science and Light Industry, Nanjing Tech University, Nanjing 211816, China; hk@njtech.edu.cn (K.H.); 202261119019@njtech.edu.cn (X.S.); 202221019052@njtech.edu.cn (Z.T.); 202261218259@njtech.edu.cn (X.H.); sunl@njtech.edu.cn (L.S.); ruiwang2013@njtech.edu.cn (R.W.); yian.gu@hotmail.com (Y.G.); xuh@njtech.edu.cn (H.X.); qyb@njtech.edu.cn (Y.Q.)

**Keywords:** alhagan, exopolysaccharide synthesis mechanism, glycosyltransferase genes, *Pantoea alhagi*, transcriptome sequencing, whole-genome sequencing

## Abstract

The exopolysaccharide (EPS) produced by *Pantoea alhagi* NX-11, referred to as alhagan, enhances plant stress resistance, improves soil properties, and exhibits notable rheological properties. Despite these benefits, the exact bio-synthetic process of alhagan by *P. alhagi* NX-11 remains unclear. This study focused on sequencing the complete genome of *P. alhagi* NX-11 and identifying an alhagan synthesis gene cluster (*LQ939_RS12550* to *LQ939_RS12700*). Gene annotation revealed that alhagan biosynthesis in *P. alhagi* NX-11 follows the Wzx/Wzy-dependent pathway. Furthermore, transcriptome analysis of *P. alhagi* NX-11 highlighted significant upregulation of four glycosyltransferase genes (*alhH*, *wcaJ*, *alhK*, and *alhM*) within the alhagan synthesis gene cluster. These glycosyltransferases are crucial for alhagan synthesis. To delve deeper into this process, two upregulated and uncharacterized glycosyltransferase genes, *alhH* and *alhK*, were knocked out. The resulting mutants, ΔalhH and ΔalhK, showed a notable decrease in EPS yield, reduced molecular weight, and altered monosaccharide compositions. These findings contribute to a better understanding of the alhagan biosynthesis mechanism in *P. alhagi* NX-11.

## 1. Introduction

In recent years, there has been growing interest in polysaccharides derived from natural sources such as plants, animals, and microorganisms [1]. These polysaccharides show great promise for use in various fields including medicine, food, materials, agriculture, and environmental protection [2,3,4,5,6,7]. Exopolysaccharides (EPSs) obtained from bacterial cultures, in particular, offer distinct advantages over those sourced from animals and plants due to their faster growth rates, simplified extraction processes, and a fermentation system more suitable for modern industrial settings [8]. These advantages lead to significantly reduced manufacturing costs. Moreover, the structure of EPSs, including factors like molecular weight and monosaccharide composition, can be customized through microbial genetic engineering to alter the properties of EPSs and expand their potential applications. However, the limited understanding of the mechanisms underlying EPS synthesis poses a challenge to the advancement of these ideas.

Currently, there are four main mechanisms known for the biosynthesis of polysaccharides in bacteria. The first mechanism involves the Wzx/Wzy-dependent pathway, where glycosyltransferases (GTs) assemble repeating units, which are then flipped into the periplasm by the flippase protein (Wzx), polymerized by the polymerase (Wzy), and transported out of the cell by outer membrane export proteins (OPX proteins) [9]. This pathway is responsible for the production of secretory branched heteropolysaccharides like xanthan and welan gum [10,11]. The second mechanism is the ATP-binding cassette (ABC) transporter-dependent pathway, where the polymer chain is assembled in the cytoplasm by various GTs and then flipped into the periplasm. Capsular heteropolysaccharides, such as lipopolysaccharides, are mainly synthesized through this pathway [12]. The third mechanism is the synthase-dependent pathway, which polymerizes a single type of monosaccharide using a specific GT domain to produce homopolysaccharides like alginic acid and bacterial cellulose [13]. Finally, the fourth mechanism is the extracellular synthesis pathway, where a single sucrase protein is involved in assembling homopolymers or specific oligosaccharides through substrate cleavage and monomer polymerization [14]. The diversity and complexity of the structure and synthetic pathway of bacterial polysaccharides have made the analysis of their synthetic mechanism a long-term project that has garnered increasing attention.

In previous studies, *P. alhagi* NX-11 was identified for its high exopolysaccharide (EPS) yield from the rhizosphere soil of sea rice. The EPS produced by NX-11, named PAPS, is a heteropolysaccharide consisting of galactose, glucose, glucosamine, glucuronic acid, and mannose [15]. Research by Sun et al. demonstrated that PAPS facilitated the colonization of *P. alhagi* NX-11 in plant roots, boosting plant resistance by enhancing antioxidant activities, suggesting its potential as a plant biostimulant [16]. However, the understanding of the synthesis mechanism of *P. alhagi* EPS, and *Pantoea* sp. EPS in general, remains limited, hindering efforts to enhance EPS synthesis efficiency and design its structure using metabolic engineering strategies. In the era of post-genomics, omics analysis (genomics, proteomics, transcriptomics, and metabolomics) has emerged as a crucial tool in biological research. For instance, Wu et al. identified the EPS synthesis locus of *Pseudomonas stutzeri* 273 through genomics, leading to the knockout of two neighboring genes responsible for GT (*eps273-H* and *eps273-I*) and a tyrosine protein kinase (*eps273-O*) in the EPS gene cluster, thus confirming their involvement in EPS biosynthesis [17]. Similarly, Padmanabhan et al. employed transcriptomics to elucidate the regulatory mechanism of EPS synthesis in *Streptococcus thermophilus* ASCC 1275, revealing that EPS synthesis and transport in this bacterium adhere to the Wzx/Wzy-dependent pathway [18]. Therefore, omics analysis holds promise as a valuable approach to uncover the pathways of polysaccharide biosynthesis.

In this research, we characterized the gene cluster responsible for alhagan synthesis in *P. alhagi* NX-11 using genomics and bioinformatics analysis. We also investigated the alhagan metabolic pathway and assessed changes in gene transcription levels during production culture with varying EPS yields through transcriptomics. Our findings identified key genes involved in alhagan synthesis, laying a foundation for the future development and application of alhagan from *P. alhagi* NX-11.

## 2. Materials and Methods

### 2.1. Strains, Plasmids and Media

The strains and plasmids used in this study are listed in Table S1, the primers used are listed in Table S2, and the media formulations used are as follows:

The seed medium formula was composed of 2% sucrose, 1% tryptone, 0.5% yeast powder and 1% NaCl. The production culture medium was composed of 6% sucrose, 1% tryptone, 0.5% NaCl and 0.1% KH_2_PO_4_. The solid production culture medium consisted of 6% sucrose, 1% tryptone, 0.5% NaCl, 0.1% KH_2_PO_4_ and 2% agar. The transcriptomic CK medium consisted of 1% tryptone, 0.5% yeast powder, 1% NaCl and 0.1% KH_2_PO_4_. The transcriptome Suc medium was composed of 4% sucrose, 1% tryptone, 0.5% yeast powder, 1% NaCl and 0.1% KH_2_PO_4_.

### 2.2. Whole-Genome Sequencing and Gene Function Annotation

*P. alhagi* NX-11 was inoculated on LB solid medium and incubated overnight at 37 °C. A single colony was then selected and transferred into LB liquid medium, followed by incubation at 37 °C and 200 rpm for 24 h. DNA was extracted from 5 mL of bacteria, and the genome of *P. alhagi* NX-11 was sequenced and assembled by Novogene Co., Ltd. (Beijing, China).

Utilizing the Illumina NovaSeq PE150 platform (Illumina, Inc., San Diego, CA, USA) for sequencing the whole genome of *P. alhagi* NX-11 resulted in a certain proportion of low-quality data within the original dataset. To ensure the accuracy and reliability of subsequent information analysis, filtering of the original data was necessary to obtain effective data. The genome reads were assembled using SMRT Link (Version 5.0.1) [19,20], and coding genes of the newly sequenced genome were predicted with GeneMarkS (Version 4.17) [21]. Subsequently, gene function was annotated using GO, KEGG, COG, NR, Pfam, TCDB, and Swiss-Prot databases.

### 2.3. Transcriptome Sequencing

The activated *P. alhagi* NX-11 strain was inoculated into the seed medium and incubated at 37 °C and 200 rpm for 12 h. Subsequently, the inoculum was transferred to CK medium and Suc medium at an inoculation rate of 4%. Three parallel groups were established for each medium and incubated in a constant-temperature shaker at 30 °C and 200 rpm for 8 h. Following incubation, the cells were harvested by centrifugation at 4 °C and 8000 rpm for 10 min, and designated as the CK group and Suc group, respectively. Each group consisted of 3 biological replicates. The samples were rapidly frozen in liquid nitrogen and stored in a −80 °C freezer for future use.

Total RNA was extracted from the sample by RNAisoPlusKit (TaKaRa Bio, Inc., Shiga, Japan). The concentration and purity of the extracted RNA were detected by Nanodrop 2000 (Thermo Fisher Scientific, Inc., Wyman Street, Waltham, MA, USA), the integrity of RNA was confirmed by agarose gel electrophoresis, and the Rin value was determined by Agilent 2100 (Agilent Technologies, Inc., Santa Clara, CA, USA) [22]. The RNA-seq library was prepared using the TruSeqTM Stranded Total RNA Library Prep Kit (Illumina, Inc., San Diego, CA, USA) and subsequently sequenced on an Illumina platform. The construction and sequencing of the RNA-Seq library was carried out by Shanghai Majorbio Bio-Pharm Technology Co., Ltd. (Shanghai, China).

### 2.4. Quantitative Real-Time PCR Validation

The RNA-seq results were validated through qRT-PCR using the StepOnePlus TM real-time PCR system (Applied Biosystem, Inc., Foster City, CA, USA). A total of 12 differential genes were selected, as described in Section 3. Total RNA was extracted following the previously described method and reverse transcribed with the PrimeScript RT premix kit (TaKaRa Bio, Inc.) as per the manufacturer’s instructions. Subsequently, the SYBR Premix Ex TaqTM II kit (TaKaRa Bio, Inc., Shiga, Japan) was utilized for the qRT-PCR process. The qRT-PCR reaction, with a final volume of 20 μL, included an initial step of 30 s at 95 °C, followed by 40 cycles of 5 s at 95 °C and 30 s at 60 °C. The 16S rRNA gene served as the internal reference gene [23], and the relative change in gene expression was determined using the 2^−ΔΔCt^ method [24].

### 2.5. Construction of Overexpression Strains

Using the *P. alhagi* NX-11 genome as a template, the *alhH* gene fragment was amplified with the primer alhH-F/alhH-R. The pKT100 plasmid served as the template, and the primer pKT100-F/pKT100-R was used to clone the pKT100 vector frame. The target gene fragment was then cloned into the pKT100 vector using In-Fusion cloning and transformed into *Escherichia coli* DH5α competent cells. The transformants were confirmed by colony PCR using the primer pKT100-CX-F/pKT100-CX-R, resulting in the recombinant plasmid pKT100-alhH. Subsequently, the recombinant plasmid pKT100-alhH was electrotransformed into *P. alhagi* NX-11, leading to the overexpression strain of the *alhH* gene, named NX-11 (pKT100-alhH). Similarly, the construction method for the overexpression strain NX-11 (pKT100-alhK) of the *alhK* gene followed the same procedure.

### 2.6. Construction of Knockout Strains

The method of *P. alhagi* NX-11 electric shock conversion was detailed in a previous study with some modifications [16]. The activated *P. alhagi* NX-11 was transferred to 37 °C LB medium and cultured overnight at 200 rpm. Subsequently, it was transferred to 100 mL of fresh LB medium with a 1% inoculation and cultured at 30 °C and 200 rpm until an OD_600_ of 0.6–0.8 was reached. The bacterial solution underwent centrifugation at 4 °C with 4600× *g* for 8 min and was washed thrice with cold deionized water. Following the removal of the supernatant, the cells were re-suspended in 1 mL of cold deionized water. Post re-suspension, the cells were divided into 10 tubes, each containing 100 μL of competent cells, and mixed with 10 μL (200 ng) of plasmid. Electroporation was then carried out using a MicroPulser 1652100 (Bio-Rad Laboratories, Inc., Hercules, CA, USA) with an electric field intensity of 20 kV/cm and a pulse time of 4 ms. The cells were promptly added to 900 mL of LB medium and incubated at 30 °C with 200 rpm for 2 h. Subsequently, they were plated on antibiotic-containing plates and positive clones were identified through colony PCR.

*P. alhagi* NX-11 is knocked out without trace based on CRISPR/Cas9 system [25,26]. Initially, the plasmid pCas was electrotransformed into NX-11 and verified, followed by electrotransformation of the recombinant plasmid pTarget-alhH into NX-11 (pCas). Verification of transformants was conducted via colony PCR using primer alhH-out-F/alhH-out-R. Positive clones were then cultured overnight in LB medium (containing Km and IPTG) at 30 °C with agitation at 200 rpm, resulting in the loss of the pTarget-alhH plasmid. Subsequently, the positive clones were cultured overnight at 37 °C with agitation at 200 rpm to lose the pCas plasmid. The resulting knockout mutant strain was named ΔalhH. The construction method for the knockout strain ΔalhK followed the same procedure.

### 2.7. Construction of alhH and alhK Gene Complement Strain

The recombinant plasmid pKT100-alhH was electrotransformed into the mutant ΔalhH to create the complementary strain ΔalhH (pKT100-alhH). Similarly, the construction method for strain ΔalhK (pKT100-alhK) followed the same procedure (Figure S4).

### 2.8. Analysis of Yield, Molecular Weight and Monosaccharide Components of EPS

The EPS yield was determined following the method described in a previous study [26]. The production culture broth of *P. alhagi* NX-11 was diluted three times with ultra-pure water, mixed with diatomite, and then subjected to vacuum filtration to remove the bacteria. Subsequently, the filtrate was concentrated to one-fifth of its original volume, and the concentrated supernatant underwent deproteinization three times using Sevag reagent (n-butanol:chloroform = 1:4, *v*/*v*). Following this, overnight precipitation of alcohol was induced by adding three times the volume of a 95% ethanol solution at 4 °C, and the yield was determined post freeze-drying.

The molecular weight of EPS was determined using high-performance gel-permeation chromatography [27]. Specifically, 5 mg of EPS was dissolved in 1 mL of distilled water, filtered through a 0.22 μm membrane for uniformity, and then analyzed using a high-performance gel-permeation chromatography (HPGPC) system with multi-angle laser light scattering (MALLS) and refractive index (RI) detection (HPGPC-MALLS-RI). The average molecular weight of EPS was determined on an Agilent 1260 HPLC system (Agilent Technologies, Inc., Santa Clara, CA, USA), coupled with a DAWN HELEOS-II laser photometer (Wyatt Technology Co., Santa Barbara, CA, USA) and utilizing both an Ultrahydrogel™ 500 gel-filtration chromatography column (7.8 × 300 mm) and an Ultrahydrogel™ linear gel-filtration chromatography column (7.8 × 300 mm) (Waters, Corp., Milford, MA, USA). The elution was carried out with a 2 mmol/L CH_3_COONH_4_ solution at 55 °C and a flow rate of 0.8 mL/min.

EPS monosaccharide composition analysis was conducted using pre-column PMP derivatization, following the methodology outlined in our previous publication [15]. Initially, a 5 mg sample was hydrolyzed with 4 mL of 2 mol/L TFA at 120 °C for 4 h. Subsequently, excess TFA in the hydrolysate was removed using methanol under vacuum. The freeze-dried hydrolysate was then derivatized with 500 μL of 0.3 mmol/L NaOH and 500 μL of PMP (methanol solution with a final concentration of 0.5 mmol/L) at 100 °C. The reaction was stopped by adding 500 μL of 0.3 mmol/L HCl, followed by the addition of ultra-pure water to a final volume of 2 mL. The resulting solution was extracted 3–5 times with an equal volume of chloroform, and the water phase was retained and filtered through a 0.22 μm membrane. Monosaccharide components were separated and analyzed using a C18 column (4.6 mm × 250 mm) and a Shimazu HPLC system (Shimadzu Co., Ltd., Kyoto, Japan) operating at a detection wavelength of 250 nm. The mobile phase consisted of 82% (*v*/*v*) 0.1 mol/L PBS (pH 6.8) and 18% (*v*/*v*) acetonitrile, with a flow rate of 0.8 mL/min at 30 °C. By comparing the retention time and peak area of standard monosaccharides, the composition and content of monosaccharides in EPS were determined.

### 2.9. Data Analysis

All results in this study were presented as the mean ± standard deviation (SD) of three independent biological replicates. One-way analysis of variance (ANOVA) was conducted using IBM SPSS Statistics 26.0 to determine statistical significance between groups (*p* < 0.05).

## 3. Results and Discussion

### 3.1. Genome Characteristics and Functional Annotation of P. alhagi NX-11

To investigate the alhagan biosynthetic pathway of *P. alhagi* NX-11, we conducted whole-genome sequencing and utilized bioinformatics analysis to construct a comprehensive genome map of *P. alhagi* NX-11 (Figure S1). The complete genome of *P. alhagi* NX-11 consists of a circular chromosome spanning 4,260,552 bp without any plasmids, with a GC content of 53.6%. This GC content falls within the established range for *Pantoea* species (52.7–60.6%) and is most similar to that of *P. alhagi* LTYR-11Z (53.4%) [28]. The genome contains 4192 annotated genes, totaling 3,711,663 bp, which represents 87.12% of the entire genome. Among these genes, 3928 are protein-coding sequences (CDS), while the genome also harbors 82 tRNA genes and 22 rRNA genes (Table 1).

Subsequently, genome-wide functional annotation and analysis of *P. alhagi* NX-11 were conducted. The COG annotation revealed that 3283 genes were annotated from the predicted genome coding region, with 358 genes associated with amino acid transport and metabolism, 354 genes with carbohydrate transport and metabolism, and 243 genes with translation, ribosome structure, and biogenesis (Figure S2A). In the GO database, 2780 genes were annotated and categorized into three main functional categories: molecular function, cellular component, and biological processes (Figure S2B). Through KEGG annotation, a total of 3806 genes were identified, including 200 genes involved in amino acid metabolism, 259 genes in carbohydrate metabolism, and genes related to membrane transport, nucleotide metabolism, and translation (Figure S2C). Furthermore, 55 genes were annotated as GTs by the CAZy database (Figure S2D).

### 3.2. Alhagan Synthesis Gene Cluster of P. alhagi NX-11

The biosynthesis pathway of EPS is intricate, involving enzymes responsible for EPS synthesis as well as those involved in the production of cell wall polysaccharides and lipopolysaccharides [29]. Genes associated with EPS synthesis can be categorized into three groups: those encoding nucleotide sugar synthesis, GTs, and polysaccharide polymerization and export. The genes responsible for EPS biosynthesis are typically organized in gene clusters, which exhibit a high level of conservation [30]. These gene clusters encode enzymes that participate in the synthesis of polysaccharides by sequentially adding sugars to lipid carriers anchored in the membrane, followed by polymerization and export [31].

**Figure 1:**
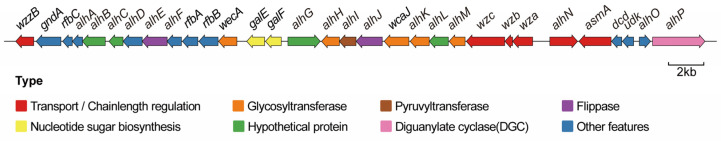
Alhagan synthesis gene cluster of *P. alhagi* NX-11.

A putative alhagan biosynthetic gene cluster (LQ939_RS12550 to LQ939_RS12700) was identified using AntiSMASH 4.0, showing 35% homology with the xanthan biosynthetic gene cluster (BGC0000800). The gene cluster spans about 39.1 kb and includes 31 putative ORFs (Figure 1). The functional annotations of the proteins encoded by genes in the alhagan gene cluster are detailed in Table 2. Specifically, genes *galE* and *galF* participate in the biosynthesis of UDP-galactose and UDP-glucose, which serve as precursors for alhagan synthesis in *P. alhagi* NX-11. Additionally, wecA, alhH, wcaJ, alhK, and alhM are predicted GTs responsible for transferring specific sugar moieties to nascent repeating units. Genes *alhE* and *alhJ* encode Wzx flip proteins involved in sugar moiety translocation, while gene *wzzB* is a chain-length-determining protein belonging to the PCP family. The gene *wzc* encodes a tyrosine protein kinase that regulates chain length, with its phosphorylation state controlled by the phosphatase wzb. Furthermore, the gene *wza* is a member of the outer membrane polysaccharide export family (OPX) [32]. The gene *alhP*, located at the end of the cluster, encodes diguanylate cyclase (DGC), which synthesizes cyclic bis(3′-5′)diguanylic acid (c-di-GMP) in cells. c-di-GMP serves as a crucial messenger controlling various bacterial cell functions such as virulence, motility, EPS biosynthesis, adhesion, secretion, biofilm formation, and cell differentiation [33].

### 3.3. Alhagan Biosynthesis Pathway of P. alhagi NX-11

**Figure 2:**
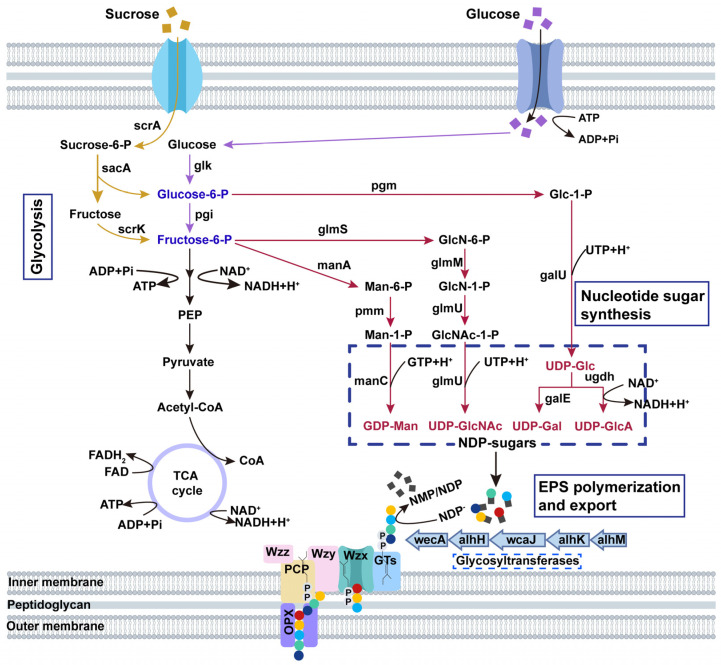
Alhagan biosynthesis pathway of *P. alhagi* NX-11. scrA: sucrose PTS permease; sacA: beta-fructofuranosidase; scrK: fructokinase; glk: glucokinase; pgi: glucose-6-phosphate isomerase; pgm: phosphoglucomutase; glmS: glutamine-fructose-6-phosphate transaminase (isomerizing); manA: mannose-6-phosphate isomerase; pmm: phosphomannomutase; manA: mannose-6-phosphate isomerase; glmM: phosphoglucosamine mutase; glmU: bifunctional UDP-N-acetylglucosamine pyrophosphorylase/glucosamine-1-phosphate N-acetyltransferase; galU: UTP-glucose-1-phosphate uridylyltransferase; ugdh: UDP-glucose 6-dehydrogenase; galE: UDP-glucose 4-epimerase.

The complexity of EPS biosynthesis is directly influenced by the diversity of EPS chemical skeleton structure. A higher complexity in EPS biosynthesis indicates a greater number of genes involved [34]. Previous studies have examined the chemical skeleton structure and components of alhagan, revealing that it primarily consists of glucose and galactose, with small amounts of glucuronic acid, glucosamine, and mannose [15]. The EPS biosynthesis process encompasses substrate uptake, nucleoside diphosphate monosaccharide (NDP-sugars) synthesis, repeat unit assembly, translocation, polymerization, and export [35]. The alhagan gene cluster of *P. alhagi* NX-11 includes five GT genes (*wecA*, *alhH*, *wcaJ*, *alhK*, and *alhM*) along with polymerization/output factors (*alhE*, *alhJ*, *wzc*, *wzb*, and *wza*). It is evident that alhagan polymerization and export in *P. alhagi* NX-11 follows the Wzx/Wzy-dependent pathway, a common pathway in microbial EPS synthesis similar to xanthan gum [36], gellan gum [37], and welan gum biosynthesis [11]. By integrating the functional analysis of genes associated with polysaccharide synthesis from genome-wide studies, a speculative schematic diagram of the Wzx/Wzy-dependent alhagan biosynthesis mechanism of *P. alhagi* NX-11 was proposed, and the metabolic pathway of *P. alhagi* NX-11 synthesizing alhagan from glucose and sucrose was depicted in Figure 2.

### 3.4. Transcriptome Sequencing and Differential Gene Enrichment Analysis

**Figure 3:**
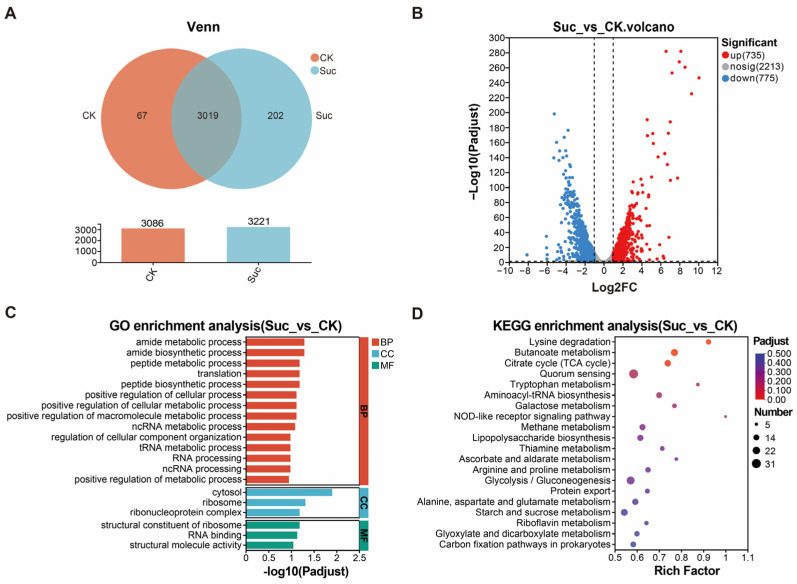
Transcriptome analysis of *P. alhagi* NX-11. (**A**) Venn plot of expression. (**B**) Volcano plot of expression difference. (**C**) Histogram of classification and enrichment significance of GO terms. (**D**) Bubble plot of enrichment significance of KEGG pathway.

To investigate global gene expression changes during sugar production in *P. alhagi* NX-11 and analyze key genes related to alhagan synthesis, we conducted transcriptome sequencing (RNA-seq) on *P. alhagi* NX-11 grown in a high EPS yield Suc medium and a low-EPS-yield CK medium. The disparity in EPS yield between these media is illustrated in Figure S3. Figure 3A displays the gene expression profiles of the CK and Suc groups, with 3086 genes expressed in CK and 3221 genes expressed in Suc. Of these, 3019 genes are expressed in both groups, 202 genes are exclusive to the Suc group, and 67 genes are unique to the CK group. In Figure 3B, upregulated genes are denoted in red, downregulated genes in blue, and genes with no significant change in gray. The Suc group exhibited 735 upregulated genes and 775 downregulated genes compared to the CK group.

The GO database was utilized for clustering and enrichment analysis of differential genes, resulting in the selection of the top 20 significantly enriched GO terms for classification and statistical analysis across three categories: biological process (BP), cellular component (CC), and molecular function (MF) (Figure 3C). The differentially expressed genes showing significant enrichment were predominantly associated with cytosol, ribosome, amide metabolism process, peptide metabolism process, polypeptide biosynthesis, cell metabolism process, and macromolecular metabolism process. Subsequently, a statistical analysis was conducted on the top 20 KEGG pathways with the most significant enrichment, spanning four categories: genetic information processing (GIP), metabolism (M), cellular process (CP), and organic system (OS) (Figure 3D). The differential genes identified between the Suc and CK groups were notably enriched in pathways such as lysine degradation, butyrate metabolism, TCA cycle, quorum sensing, tryptophan metabolism, galactose metabolism, lipopolysaccharide biosynthesis, arginine and proline metabolism, glycolysis/gluconeogenesis pathway, protein output, alanine, aspartic acid, and glutamate metabolism, as well as starch and sucrose metabolism.

Therefore, the impact of EPS yield on *P. alhagi* NX-11 primarily focused on pathways associated with amino acid metabolism and biosynthesis, ribosome and carbohydrate metabolism. Specifically, the biosynthesis and metabolism of alanine, aspartic acid, and glutamic acid were closely linked to extracellular polysaccharide biosynthesis. This process involved sucrose metabolism, the glycolysis pathway, galactose metabolism, and the TCA cycle.

### 3.5. Analysis of Alhagan Biosynthesis Genes and qPCR Verification

**Figure 4:**
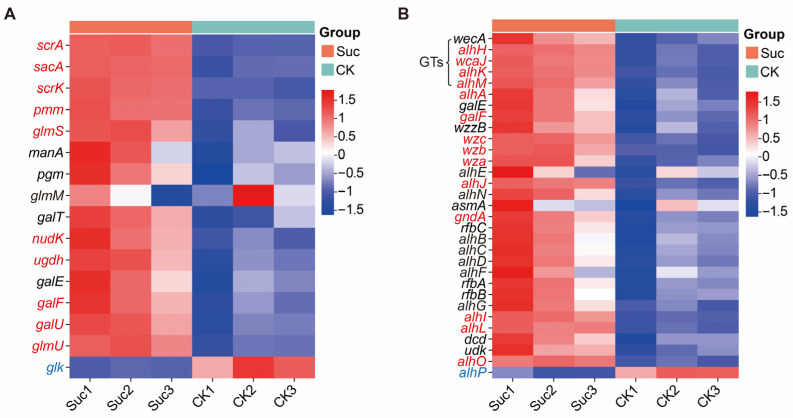
Transcription heat map of alhagan synthesis. (**A**) Transcription heat map of nucleotide sugar synthesis. (**B**) Transcription heat map of alhagan gene cluster. Red represents significantly upregulated genes, blue represents significantly downregulated genes, and black represents no significant difference.

In order to investigate changes in gene expression levels during alhagan synthesis in *P. alhagi* NX-11, a differential analysis of alhagan biosynthesis genes was conducted. The results are detailed in Figure 4 and Table S3. Alhagan synthesis encompasses nucleotide sugar synthesis, polysaccharide repeat unit assembly, and EPS polymerization and secretion. Nucleotide sugar synthesis involves the PTS transport system, amino sugar, and nucleotide sugar metabolism. In comparison to the CK group, the Suc group exhibited significant upregulation of *pmm*, *ugdh*, *galU*, *galF*, *nudK*, *scrK*, *scrA*, *sacA*, *glmU*, and *glmS* genes, while the *glk* gene was notably downregulated. Extracellular sucrose is transformed into sucrose 6-phosphate by sucrose PTS permease encoded by *scrA* and further metabolized into glucose 6-phosphate and fructose by beta-fructofuranosidase encoded by *sacA*. Fructokinase encoded by *scrK* facilitates the conversion of fructose to fructose-6-phosphate, ultimately leading to the synthesis of nucleotide sugar precursor through glucose-6-phosphate and fructose-6-phosphate metabolism. The significant downregulation of the *glk* gene suggests a weakened pathway for glucose synthesis from glucose to 6-phosphoglucose in sucrose metabolism, aligning with the previously hypothesized alhagan synthesis pathway from sucrose.

The assembly of repeat units primarily relies on the activity of GTs. Within the alhagan synthesis gene cluster, five GTs were identified, with *alhH*, *wcaJ*, *alhK*, and *alhM* showing significant upregulation in expression. Additionally, the *wecA* gene exhibited a slight increase in expression, highlighting the crucial role of GTs in polysaccharide synthesis. In the process of alhagan polymerization and secretion, the upregulation of *alhJ*, *wzc*, *wzb*, and *wza* genes significantly contributed to alhagan synthesis. Furthermore, the analysis of other genes in the alhagan gene cluster revealed significant upregulation in the expression of *gndA*, *alhA*, *alhI*, *alhL*, and *alhO* genes, while the *alhP* gene was notably downregulated. Notably, *alhP* influences the synthesis of intracellular c-di-GMP, a second messenger in the cell that regulates the production of EPSs such as cellulose [38], alginate [39] and xanthan gum [40].

Twelve differential genes were chosen from the alhagan synthesis pathway and alhagan gene cluster for qRT-PCR analysis. The results of the qRT-PCR analysis were in agreement with the RNA-seq data (Table S4), confirming the validity of the RNA-seq data.

### 3.6. Characterization of the GT Genes alhH and alhK

Among the enzymes required for EPS biosynthesis, GTs have garnered significant attention due to their sugar specificity, which plays a crucial role in determining the composition and structural features of EPSs [41]. Priming GTs are responsible for linking the initial sugar of the repeating unit to the lipid carrier [35]. These priming GTs typically exhibit high homology, unlike other GTs that are often more unique or less homologous [42]. Despite the sequencing of numerous EPS gene clusters, only a limited number of GTs have been biochemically characterized [43]. Within the *P. alhagi* NX-11 alhagan gene cluster, five GTs were predicted. Transcriptional analysis revealed significant upregulation (by 12, 16, 13, and 7 times) of the expressions of *alhH*, *wcaJ*, *alhK*, and *alhM* during alhagan synthesis. Notably, the gene *wcaJ* functions as the priming GT, facilitating the attachment of the first sugar (glucose-1-P) to the lipid carrier undecylpropenyl phosphate (Und-P). Previous studies involving an EPS-deficient strain, ΔpapD (where *papD* corresponds to *wcaJ*), elucidated the importance of PAPS in enhancing rice stress resistance and specific root colonization in *P. alhagi* NX-11 [16]. Furthermore, the gene *alhM* shares a 99% similarity with the gene *tuaG* (*B1H58_14500*) in strain *P. alhagi* LTYR-11Z. Zhang et al. created a mutant strain, ΔtuaG, by deleting the gene *tuaG*, which resulted in a significant decrease in EPS yield [23]. Consequently, our investigation focused on the remaining two GT genes, *alhH* and *alhK*.

**Figure 5:**
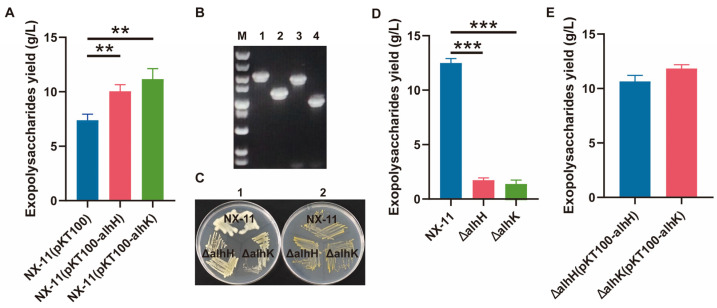
Effects of overexpression, knockout and complementation of *alhH* and *alhK* genes on EPS yield. (**A**) The EPS yield of strains NX-11 (pKT100), NX-11 (pKT100-alhH) and NX-11 (pKT100-alhK). (**B**) Nucleic acid electropherograms of mutant strains ΔalhH and ΔalhK. M: 5000 bp DNA maker, 1 and 3: NX-11, 2: ΔalhH, 4: ΔalhK. The primers used in 1 and 2 are alhH-out-F/alhH-out-R, 3 and 4 are alhK-out-F/alhK-out-R. (**C**) The colony morphology of strains ΔalhH, ΔalhK and *P. alhagi* NX-11 on solid medium. 1: solid production culture medium, 2: solid LB medium. (**D**) The EPS yield of strains ΔalhH, ΔalhK and *P. alhagi* NX-11. (**E**) The EPS yield of complementary strains ΔalhH (pKT100-alhH) and ΔalhK (pKT100-alhK). **, *p* ≤ 0.01; ***, *p* ≤ 0.001.

The genes *alhH* and *alhK* were overexpressed to investigate their impact on EPS yield. Results in Figure 5A show that the overexpressed strains NX-11 (pKT100-alhH) and NX-11 (pKT100-alhK) exhibited significantly higher EPS yields compared to the control, suggesting a role of these genes in alhagan synthesis. Subsequently, genes *alhH* and *alhK* were knocked out individually to further elucidate their effects on alhagan synthesis, as depicted in Figure 5B. Colony morphology comparisons in solid production culture medium and solid LB medium, along with EPS yield in liquid production culture medium, were conducted for ΔalhH, ΔalhK, and *P. alhagi* NX-11, with results shown in Figure 5C,D. While the colony morphology of ΔalhH, ΔalhK, and *P. alhagi* NX-11 in solid LB medium did not differ significantly, the mutants ΔalhH and ΔalhK displayed fine, smooth colony morphology in solid production culture medium, contrasting with *P. alhagi* NX-11. Notably, the EPS secretion of ΔalhH and ΔalhK mutants decreased significantly to 1.73 g/L and 1.39 g/L, respectively. Moreover, Figure 5E demonstrates a significant increase in EPS yield for complementing strains ΔalhH (pKT100-alhH) and ΔalhK (pKT100-alhK).

**Figure 6:**
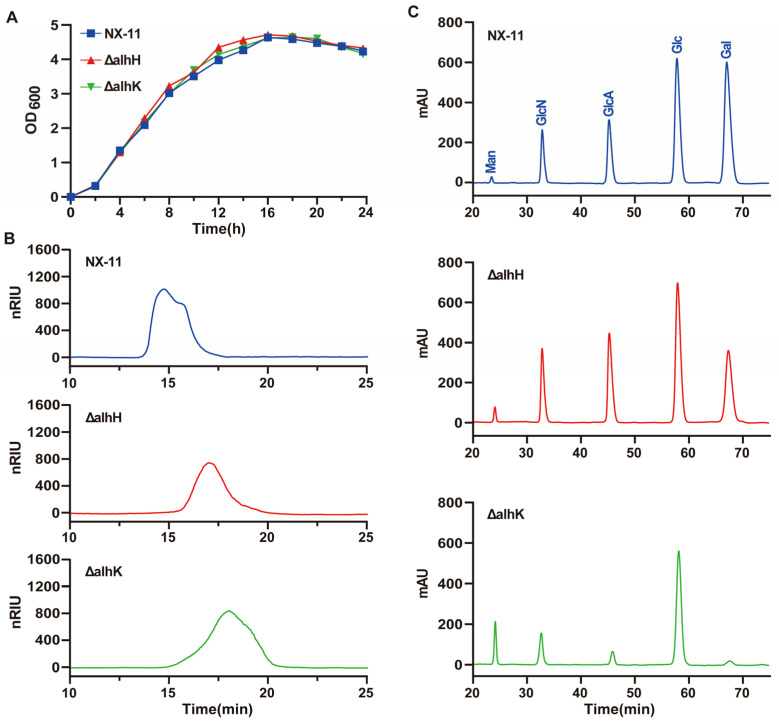
Differences in growth curves, molecular weight and monosaccharide composition of strains ΔalhH, ΔalhK and *P. alhagi* NX-11. (**A**) The growth curve of ΔalhH, ΔalhK and *P. alhagi* NX-11. (**B**) The EPS molecular weight of ΔalhH, ΔalhK and *P. alhagi* NX-11. (**C**) The EPS monosaccharide component of ΔalhH, ΔalhK and *P. alhagi* NX-11.

The growth curve, EPS molecular weight, and monosaccharide compositions of strains ΔalhH, ΔalhK, and *P. alhagi* NX-11 were further analyzed. Figure 6A illustrates that the deletion of GT genes *alhH* and *alhK* did not impact the growth of the strains. Both ΔalhH and ΔalhK exhibited similar growth curves to *P. alhagi* NX-11, reaching a stable phase at around 16 h with no significant differences observed. However, there were notable changes in the molecular weight and monosaccharide compositions of EPS in strains ΔalhH and ΔalhK compared to *P. alhagi* NX-11. In Figure 6B, it is evident that the deletion of *alhH* and *alhK* genes not only decreased the EPS yield but also significantly reduced the molecular weight. The GPC spectrum of alhagan from *P. alhagi* NX-11 displayed a broad peak at 15 min, whereas the GPC spectrum of alhagan from ΔalhH and ΔalhK showed peaks at 17 and 18 min, respectively, indicating a substantial decrease in molecular weight. Moreover, the deletion of *alhH* and *alhK* genes influenced the monosaccharide composition of EPS. While all strains contained glucose, galactose, mannose, glucosamine, and glucuronic acid in their EPS compositions (Figure 6C), the relative proportions varied significantly. Specifically, compared to *P. alhagi* NX-11, the galactose content was notably reduced in the EPS of the ΔalhH strain, while both galactose and glucuronic acid contents were significantly lower in the EPS of the ΔalhK strain.

The results indicate that the overexpression of genes *alhH* and *alhK* is associated with increased alhagan synthesis. Conversely, the deletion of these genes results in a notable decrease in both EPS yield and molecular weight, as well as significant changes in the monosaccharide components of EPS. Therefore, it can be concluded that the GT genes *alhH* and *alhK* are crucial for alhagan synthesis.

## 4. Conclusions

Our results provide preliminary insights into the mechanism of alhagan biosynthesis in *P. alhagi* NX-11 and identify key genes involved in this process. In this study, we sequenced the entire genome of *P. alhagi* NX-11. Through functional analysis of the whole genome, we identified an alhagan synthesis gene cluster and predicted the pathway for alhagan biosynthesis. Furthermore, we analyzed gene expression levels during alhagan synthesis using transcriptomics. Two GT genes, *alhH* and *alhK*, which were significantly upregulated within the alhagan gene cluster, were subsequently knocked out. The EPS yield and molecular weight in the mutants ΔalhH and ΔalhK were significantly reduced, and the monosaccharide compositions of these mutants were also markedly altered. These findings indicate that the GT genes *alhH* and *alhK* are crucial for alhagan synthesis. This study lays a foundation for the development and utilization of alhagan derived from *P. alhagi* NX-11.

## Figures and Tables

**Table 1:** *P. alhagi* NX-11 genome characteristics.

Attribute	Values
Genome Size (bp)	4,260,552
GC Content (%)	53.6
Gene Number	4192
Gene Length (bp)	3,711,663
CDS (protein)	3928
tRNA genes	82
rRNA genes (5S, 16S, 23S)	22

**Table 2:** Functional annotation of alhagan-synthesis-gene-cluster-encoded protein in *P. alhagi* NX-11.

Gene ID	Length (aa)	Function	Rename
*LQ939_RS12550*	340	LPS O-antigen chain length determinant protein	*wzzB*
*LQ939_RS12555*	469	NADP-dependent phosphogluconate dehydrogenase	*gndA*
*LQ939_RS12560*	183	dTDP-4-dehydrorhamnose 3,5-epimerase	*rfbC*
*LQ939_RS12565*	193	DapH/DapD/GlmU-related protein	*alhA*
*LQ939_RS12570*	434	hypothetical protein	*alhB*
*LQ939_RS12575*	266	hypothetical protein	*alhC*
*LQ939_RS12580*	363	EpsG family protein	*alhD*
*LQ939_RS12585*	475	oligosaccharide flippase family protein	*alhE*
*LQ939_RS12590*	274	NAD(P)-dependent oxidoreductase	*alhF*
*LQ939_RS12595*	293	glucose-1-phosphate thymidylyltransferase	*rfbA*
*LQ939_RS12600*	363	dTDP-glucose 4,6-dehydratase	*rfbB*
*LQ939_RS12605*	349	UDP-N-acetylglucosamine-undecaprenyl-phosphate N-acetylglucosaminephosphotransferase	*wecA*
*LQ939_RS12610*	338	UDP-glucose 4-epimerase	*galE*
*LQ939_RS12615*	299	UTP-glucose-1-phosphate uridylyltransferase	*galF*
*LQ939_RS12620*	612	hypothetical protein	*alhG*
*LQ939_RS12625*	336	glycosyltransferase	*alhH*
*LQ939_RS12630*	316	polysaccharide pyruvyl transferase family protein	*alhI*
*LQ939_RS12635*	494	MOP flippase family protein	*alhJ*
*LQ939_RS12640*	467	undecaprenyl-phosphate glucose phosphotransferase	*wcaJ*
*LQ939_RS12645*	361	glycosyltransferase	*alhK*
*LQ939_RS12650*	360	hypothetical protein	*alhL*
*LQ939_RS12655*	302	glycosyltransferase	*alhM*
*LQ939_RS12660*	726	tyrosine-protein kinase	*wzc*
*LQ939_RS12665*	145	protein tyrosine phosphatase	*wzb*
*LQ939_RS12670*	377	polysaccharide export protein	*wza*
*LQ939_RS12675*	525	TerC family protein	*alhN*
*LQ939_RS12680*	608	outer membrane assembly protein AsmA	*asmA*
*LQ939_RS12685*	194	dCTP deaminase	*dcd*
*LQ939_RS12690*	214	uridine kinase	*udk*
*LQ939_RS12695*	214	phosphatase PAP2 family protein	*alhO*
*LQ939_RS12700*	992	diguanylate cyclase	*alhP*

## Data Availability

The datasets supporting the conclusion of this article are included in the article and Supplementary Materials. The GenBank accession number for the genome sequence of strain *Pantoea alhagi* NX-11 is CP097983.1.

## Supplementary Materials

The following supporting information can be downloaded at https://www.mdpi.com/article/10.3390/microorganisms12102016/s1: Table S1: Strains and plasmids used in this study; Table S2: Primers used in this study; Table S3: Analysis of differences in expression of synthetic genes in alhagan; Table S4: qPCR verification results of 12 differentially expressed genes; Figure S1: The complete genome map of *P. alhagi* NX-11; Figure S2: Genome-wide functional annotation of *P. alhagi* NX-11; Figure S3: Difference in EPS yield of *P. alhagi* NX-11 in CK medium and Suc medium; Figure S4: Growth curve, EPS molecular weight and monosaccharide composition analysis of complementary strains ΔalhH (pKT100-alhH) and ΔalhK (pKT100-alhK).
