# Supplementary material for: Genomic and Transcriptomic Analyses Identify Two Key Glycosyltransferase Genes alhH and alhK of Exopolysaccharide Biosynthesis in Pantoea alhagi NX-11
Source: Microorganisms. 2024 Oct 5;12(10):2016. doi: 10.3390/microorganisms12102016 (PMC11509785; doi:10.3390/microorganisms12102016)
Supplement: Supplementary file 1 [file microorganisms-12-02016-s001.zip › microorganisms-3224087-supplementary.pdf]

Genomic and Transcriptomic Analyses Identify Two Key Glycosyltransferase Genes *alhH* and *alhK* of Exopolysaccharide Biosynthesis in *Pantoea alhagi* NX-11

Kun He, Xiaolong Shi, Zhongming Tao, Xing Hu, Liang Sun, Rui Wang, Yian Gu, Hong Xu, Yibin Qiu and Peng Lei *

State Key Laboratory of Materials-Oriented Chemical Engineering, College of Food Science and Light Industry, Nanjing Tech University, Nanjing 211816, China; hk@njtech.edu.cn (K.H.); 202261119019@njtech.edu.cn (X.S.); 202221019052@njtech.edu.cn (Z.T.); 202261218259@njtech.edu.cn (X.H.); sunl@njtech.edu.cn (L.S.); ruiwang2013@njtech.edu.cn (R.W.); yian.gu@hotmail.com (Y.G.); xuh@njtech.edu.cn (H.X.); qyb@njtech.edu.cn (Y.Q.)
* Correspondence: lei-peng@njtech.edu.cn; Tel.: +86-25-58139433

Table S1. Strains, and plasmids used in this study

Strains or plasmids	Description	Reference or source
E. coli		
DH5α	Host for cloning	This lab
P. alhagi		
NX-11	Wild type strain	This study
NX-11(pCas)	NX-11 harboring pCas	This study
NX-11(pKT100)	NX-11 harboring pKT100	This study
NX-11(pKT100-alhH)	NX-11 harboring pKT100-alhH	This study
NX-11(pKT100-alhK)	NX-11 harboring pKT100-alhK	This study
ΔalhH	*alhH* deleted in NX-11	This study
ΔalhK	*alhK* deleted in NX-11	This study
ΔalhH (pKT100-alhH)	Mutant ΔalhH harboring pKT100-alhH	This study
ΔalhK (pKT100-alhK)	Mutant ΔalhK harboring pKT100-alhK	This study
Plasmids		
pCas	CRISPR-Cas9 system plasmid, Km^r	
pTarget	CRISPR-Cas9 system plasmid, Str^r	
pTarget-alhH	Construct used for in-frame deletion of *alhH*	This study
pTarget-alhK	Construct used for in-frame deletion of *alhK*	This study
pKT100	Expression vector, p15A replicon, Km^r	
pKT100-alhH	*alhH* in pKT100	This study
pKT100-alhK	*alhK* in pKT100	This study

Table S2. Primers used in this study

Primer	Sequence (5'-3')
pKT100-F	TTTAGCTTCCTTAGCTCCTGAAAATCTCGATAAC
pKT100-R	ACTGGCCGTCGTTTTACAACGTCGTGAC
alhH-F	CAGGAGCTAAGGAAGCTAAAACTAGTATGATTGAAGAGCCAACGATTC
alhH-R	GTTGTAAAACGACGGCCAGTGGATCCTCATGAGGCCCGTAGTTTTAAG
alhK-F	CAGGAGCTAAGGAAGCTAAAACTAGTATGAAAATCCTCCATGCTGC
alhK-R	GTTGTAAAACGACGGCCAGTGGATCCTCAACGTTTCAGTATGTCGG
pKT100-CX-F	ACGGAAGATCACTTCGCAGA
pKT100-CX-R	CATCAGCGCCATTCGCCATTC
pTarget-F	ACTAGTATTATACCTAGGACTGAGCTAGCTGTCAAGGAT
pTarget-R	TGAATTCTCTAGAGTCGACCTGCAGAAGCT
pTarget-CX-F	ACCGTATTACCGCCTTTGAGTGAGCTG
pTarget-CX-R	GAGTCGATACTTCGGCGATCACCGCTTC
alhH-sg-F	GTCCTAGGTATAATACTAGTATTATTCATACCGCCAATGGGTTTTAGAGCTAGAAATAG
alhH-sg-R	CGTCTCTTCGATATCTTTACGGATCATAAAAAAAGCACCGACTCGGTGCC
alhH-up-F	GGCACCGAGTCGGTGCTTTTTTTATGATCCGTAAAGATATCGAAGAGACG
alhH-up-R	TTTTCCGCCAGCGTGATATCCAGGTTAATTGAATCAACAGCTTCCTGAACAT
alhH-dn-F	ATGTTCAGGAAGCTGTTGATTCAATTAACCTGGATATCACGCTGGCGGAAA
alhH-dn-R	AGCTTCTGCAGGTCGACTCTAGAGAATTCGAGCTGAGCGGCATCAAACAGTCGCTACT
alhH-out-F	CAGTCGGCCCAGATCATTCGCCAGCATACAAACCTGACGA
alhH-out-R	TACCAGCAACAACTGCTATCACCAGCTAAACGGCACCGGT
alhK-sg-F	GTCCTAGGTATAATACTAGTAATCAAATTCAAGCCCGACGGTTTTAGAGCTAGAAATAG
alhK-sg-R	TACCGGCCGGGAACCAGGGACGAAAAAGCACCGACTCGGTGCC
alhK-up-F	GGCACCGAGTCGGTGCTTTTTCGTCCCTGGTTCCCGGCCGGTA
alhK-up-R	ATTGATTTCCTTTTCGACCCTTTTGATAACTGCCCTTCTCATCC
alhK-dn-F	GGATGAGAAGGGCAGTTATCAAAAGGGTCGAAAAGGAAATCAAT
alhK-dn-R	TGCAGGTCGACTCTAGAGAATTCAGGTAATCAAAATACTTAATGCCCA

alhK-out-F	GGTATTGACGTAGATAACTTCCGTA
alhK-out-R	ACCGATCGCCACAATCCCAAACCAC
16sRNA-RT-F	AGCTGGTCTGAGAGGATGAC
16sRNA-RT-R	GTAACGTCAATGAACCAGGT
pmm-RT-F	GCTGATTACGGCGCTGGTTA
pmm-RT-R	CCATCGTTACGGCTGAATTG
galU-RT-F	CAGATCATGGTAGAGCCGGT
galU-RT-R	TAGCCAGCAGCGGCCAAATA
galF-RT-F	TACAATCCATTTGTCCGCCG
galF-RT-R	GTATCTTCAAAGCGCGCAAC
alhH-RT-F	TTACAGACGCAGCACCAGCT
alhH-RT-R	TGCCGCATCAACTCTTCCAG
alhJ-RT-F	GATTGGGTGCCTCATTAGGA
alhJ-RT-R	CTGAACAGCTAAGAGTACCG
alhK-RT-F	GGAGTATACCGGCTGGCTTA
alhK-RT-R	AGTAAGGAGTCAGAGTCACC
wzc-RT-F	TCAGGCCAGCGCGTACTGTT
wzc-RT-R	ACTTCTGACTCATCAGCAGT
wza-RT-F	CCGCGCAATGACGAACTGAA
wza-RT-R	GCTGATAGATATTCGCGACC
alhP-RT-F	TATGGTTTTGGGGCCGCACT
alhP-RT-R	AGACCACCAGAGCGCGACAA
glk-RT-F	GACACGTTGATTTCGCTCCA
glk-RT-R	GCGCGACGACAGTCGATACA
sacA-RT-F	GTACCGTCGGAAGAGTATGA
sacA-RT-R	TCACGCACATGGCCGCTGTA
glmU-RT-F	GTGCTGCAGGCTGAGCAATT
glmU-RT-R	ACAATGCGTCCATAGCCGGT

Table S3. Analysis of differences in expression of synthetic genes in alhagan

Gene ID	Gene name	Log_2FC	P adjust
Nucleotide sugar biosynthesis			
LQ939_RS02430	*glmM*	-0.850726653	6.14E-07
LQ939_RS05310	*pmm*	2.140445516	5.43E-47
LQ939_RS06205	*pgm*	0.288547225	0.073484438
LQ939_RS06460	*galT*	0.261611948	0.248560367
LQ939_RS09710	*manA*	0.349336627	0.152864487
LQ939_RS10815	*ugdh*	1.223730451	1.44E-13
LQ939_RS10820	*galU*	1.56994264	1.75E-20
LQ939_RS12610	*galE*	0.756527362	1.74E-05
LQ939_RS12615	*galF*	1.256817811	3.73E-14
LQ939_RS13915	*glk*	-1.466971154	4.09E-18
LQ939_RS14170	*nudK*	1.919491026	3.06E-05
LQ939_RS17825	*scrK*	6.441200215	7.06E-146
LQ939_RS17835	*scrA*	5.234201785	2.63E-159
LQ939_RS17840	*sacA*	3.144556171	3.95E-74
LQ939_RS19575	*glmU*	1.581122128	4.47E-21
LQ939_RS19580	*glmS*	1.160547278	1.38E-11
EPS synthesis gene cluster			
LQ939_RS12550	*wzzB*	0.264230212	0.081027665
LQ939_RS12555	*gndA*	1.163049181	5.42E-11
LQ939_RS12560	*rfbC*	0.62190011	0.001333219
LQ939_RS12565	*alhA*	1.027072936	1.10E-06
LQ939_RS12570	*alhB*	0.430385784	0.018156029
LQ939_RS12575	*alhC*	0.574534144	0.002677138
LQ939_RS12580	*alhD*	0.302638025	0.078410778
LQ939_RS12585	*alhE*	-0.411007281	0.012099329
LQ939_RS12590	*alhF*	0.111176599	0.631839501
LQ939_RS12595	*rfbA*	0.90483086	3.45E-06
LQ939_RS12600	*rfbB*	0.346972859	0.055976212
LQ939_RS12605	*wecA*	0.453936854	0.008023797
LQ939_RS12610	*galE*	0.756527362	1.74E-05
LQ939_RS12615	*galF*	1.256817811	3.73E-14
LQ939_RS12620	*alhG*	0.876473186	7.75E-06
LQ939_RS12625	*alhH*	2.691475092	1.62E-62
LQ939_RS12630	*alhI*	3.457527688	1.89E-58
LQ939_RS12635	*alhJ*	2.796777935	2.57E-53

LQ939_RS12640	*wcaJ*	3.083113361	6.18E-63
LQ939_RS12645	*alhK*	2.799690733	5.83E-37
LQ939_RS12650	*alhL*	2.624008051	2.54E-48
LQ939_RS12655	*alhM*	1.833830633	2.91E-24
LQ939_RS12660	*wzc*	2.16749265	1.22E-41
LQ939_RS12665	*wzb*	2.474316318	9.04E-14
LQ939_RS12670	*wza*	1.016333756	5.37E-07
LQ939_RS12675	*alhN*	0.566647757	0.011916991
LQ939_RS12680	*asmA*	-0.581701779	0.000146038
LQ939_RS12685	*dcd*	0.130435158	0.569420541
LQ939_RS12690	*udk*	0.478191989	0.00561666
LQ939_RS12695	*alhO*	1.662875888	1.33E-05
LQ939_RS12700	*alhP*	-1.722519525	4.66E-26

Table S4. qPCR verification results of 12 differentially expressed genes

Group	Gene ID	Gene name	Log_2FC(RNA-seq)	-ΔΔCt(qPCR)
Suc vs CK	LQ939_RS05310	*pmm*	2.140445516	2.37
	LQ939_RS10820	*galU*	1.56994264	1.23
	LQ939_RS12615	*galF*	1.256817811	1.56
	LQ939_RS12625	*alhH*	2.691475092	2.14
	LQ939_RS12635	*alhJ*	2.796777935	2.63
	LQ939_RS12645	*alhK*	2.799690733	2.48
	LQ939_RS12660	*wzc*	2.16749265	1.69
	LQ939_RS12670	*wza*	1.016333756	0.82
	LQ939_RS12700	*alhP*	-1.722519525	-1.59
	LQ939_RS13915	*glk*	-1.466971154	-1.21
	LQ939_RS17840	*sacA*	3.144556171	2.86
	LQ939_RS19575	*glmU*	1.581122128	1.89

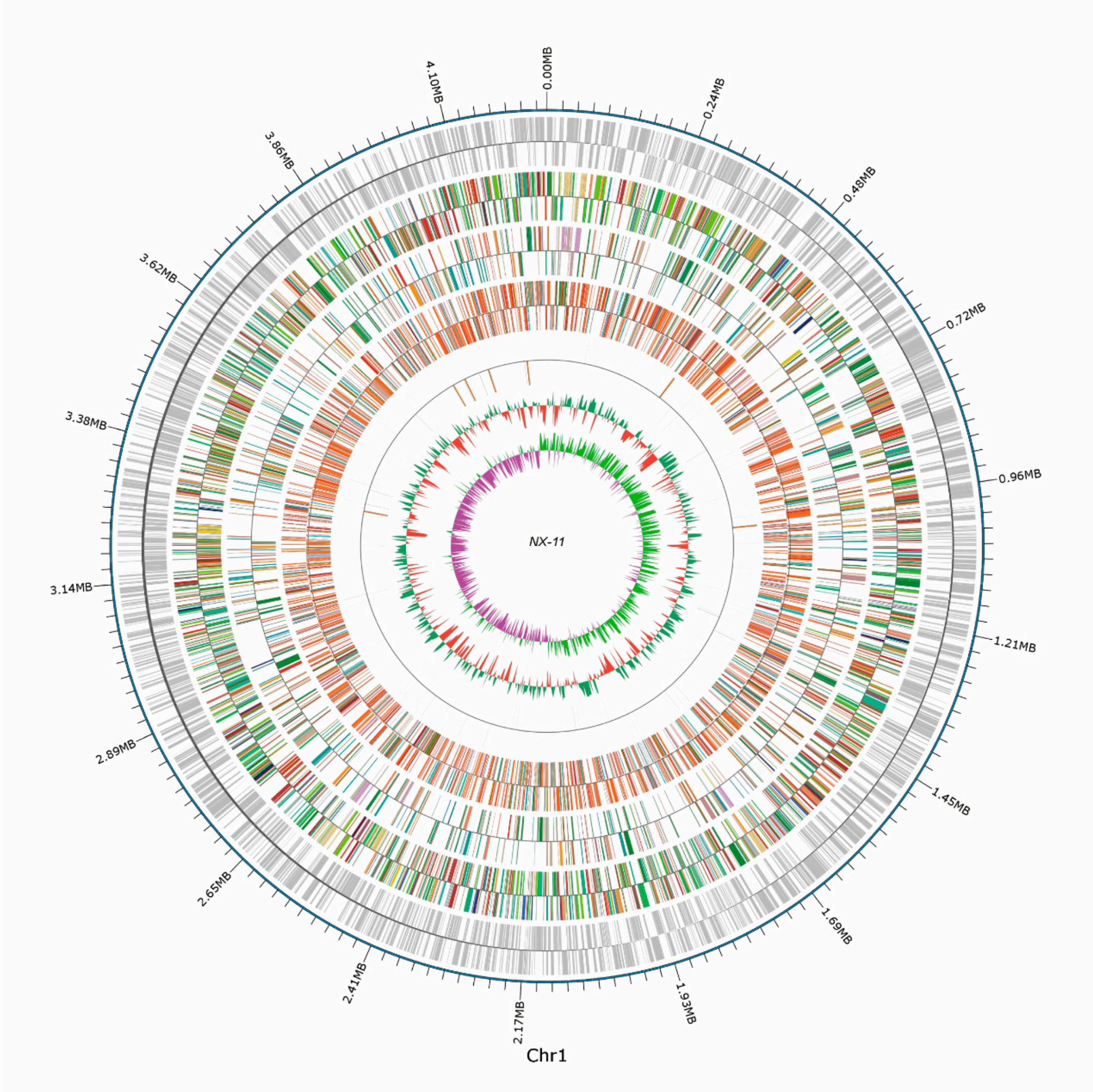

Figure S1. The complete genome map of *P. alhagi* NX-11. The outermost circle is the location coordinate of the genome sequence, which is the coding gene, the result of gene functional annotation, ncRNA, genomic GC content and genomic GC skew value from outside to inside, respectively. Genomic GC content: the inward red part indicates that the GC content in this region is lower than the average GC content of the whole genome, while the outward green part is the opposite, and the higher the peak value is, the greater the difference between the GC content and the average GC content is. The GC skew value of the genome: the inward pink part indicates that the content of G is lower than that of C, while the outward light green part is the opposite.

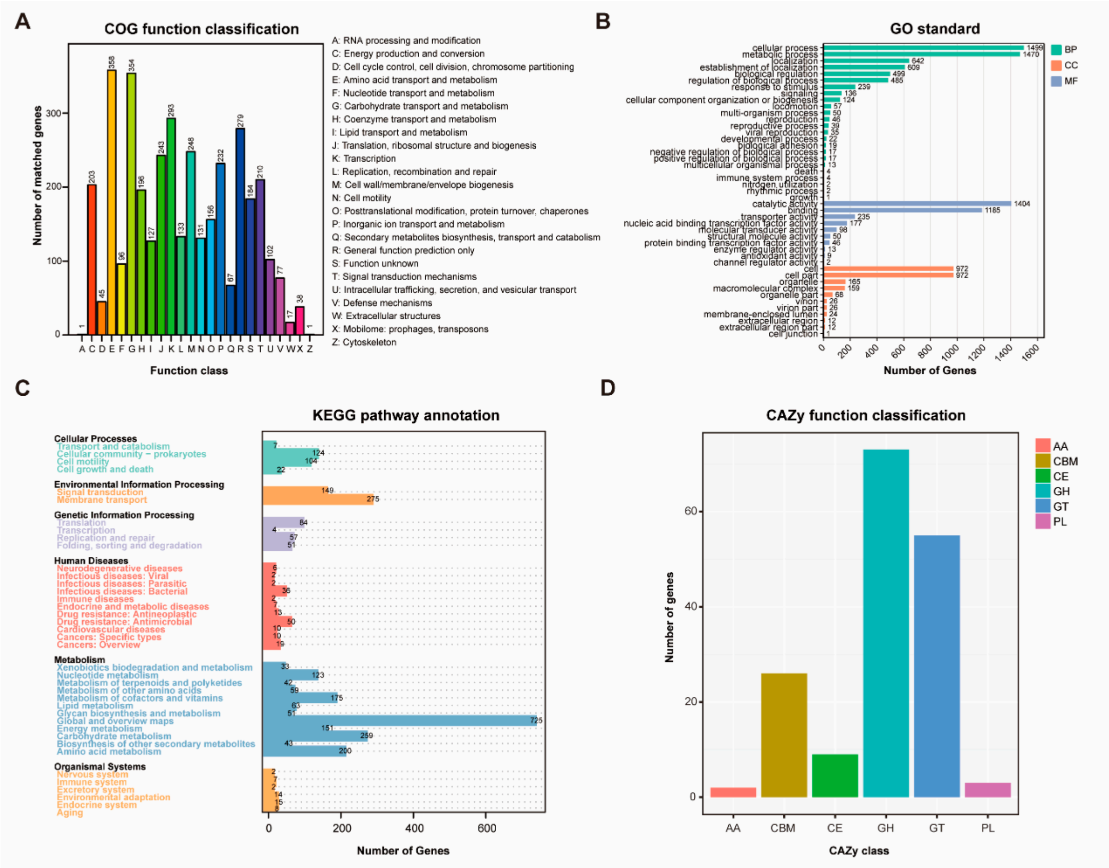

Figure S2. Genome-wide functional annotation of *P. alhagi* NX-11. (A) GOC functional classification and annotation. (B) GO functional classification and annotation. (C) KEGG pathway annotation. (D) CAzy functional classification.

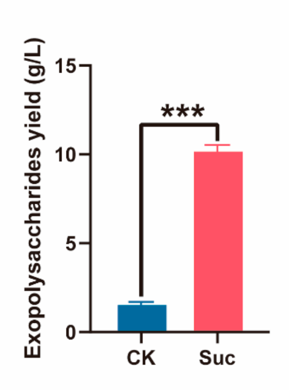

Figure S3. Difference of EPS yield of *P. alhagi* NX-11 in CK medium and Suc medium. ***, $P \leq 0.001$.

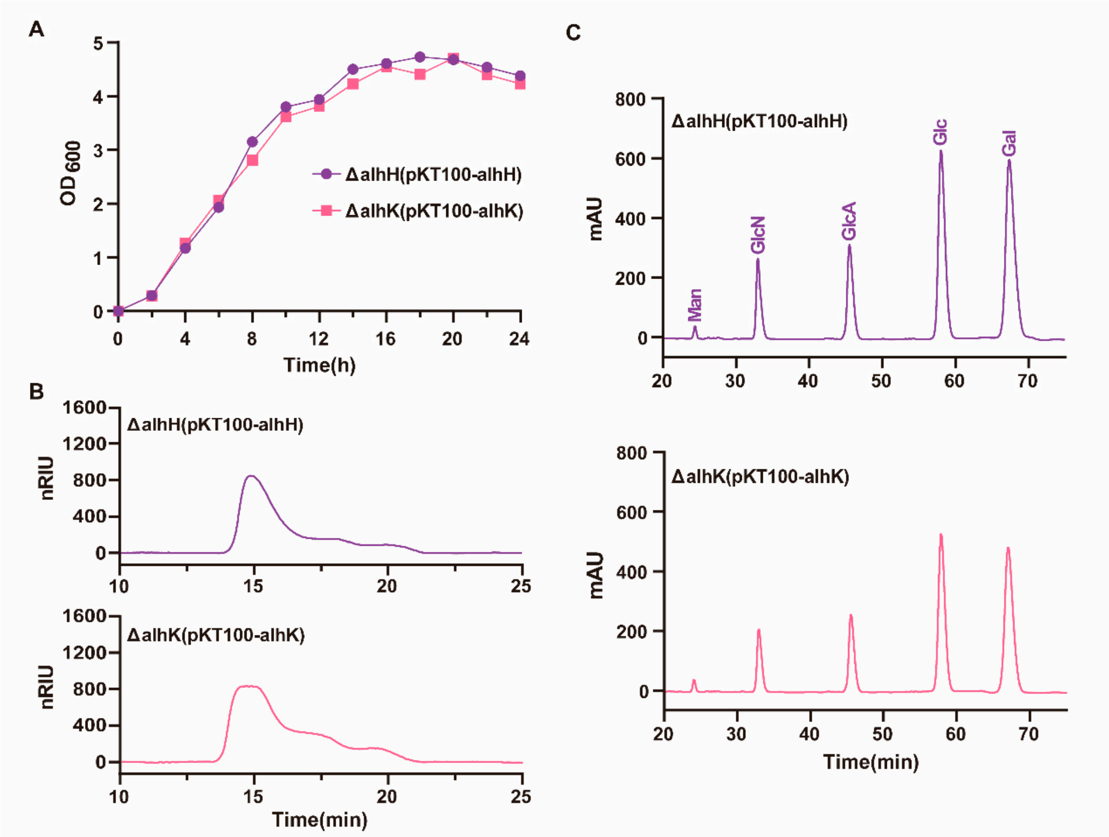

Figure S4. Growth curve, EPS molecular weight and monosaccharide composition analysis of complementary strains ΔalhH (pKT100-alhH) and ΔalhK (pKT100-alhK). (A) The growth curve of ΔalhH (pKT100-alhH) and ΔalhK (pKT100-alhK). (B) The EPS molecular weight of ΔalhH (pKT100-alhH) and ΔalhK (pKT100-alhK). (C) The EPS monosaccharide component of ΔalhH (pKT100-alhH) and ΔalhK (pKT100-alhK).
